# Chromosomal evolution of *Escherichia coli* for the efficient production of lycopene

**DOI:** 10.1186/1472-6750-13-6

**Published:** 2013-01-28

**Authors:** Yun-Yan Chen, Hong-Jie Shen, Yan-Yan Cui, Shang-Guang Chen, Zhi-Ming Weng, Ming Zhao, Jian-Zhong Liu

**Affiliations:** 1 Biotechnology Research Center and MOE Key Laboratory of Bioinorganic and Synthetic Chemistry, School of Life Science, Sun Yat-Sen University, Guangzhou, 510275, P. R. China; 2 Medical Imaging Center, Cancer Center and State Key Laboratory of Oncology in South China, Sun Yat-Sen University, Guangzhou, 510060, P. R. China

**Keywords:** Lycopene, *Escherichia coli*, Chemically induced chromosomal evolution, Metabolic engineering

## Abstract

**Background:**

Plasmid-based overexpression of genes has been the principal strategy for metabolic engineering. However, for biotechnological applications, plasmid-based expression systems are not suitable because of genetic instability, and the requirement for constant selective pressure to ensure plasmid maintenance.

**Results:**

To overcome these drawbacks, we constructed an *Escherichia coli* lycopene production strain that does not carry a plasmid or an antibiotic marker. This was achieved using triclosan-induced chromosomal evolution, a high gene copy expression system. The engineered strain demonstrated high genetic stability in the absence of the selective agent during fermentation. The replacement of native *appY* promoter with a T5 promoter, and the deletion of the *iclR* gene in *E. coli* CBW 12241 further improved lycopene production. The resulting strain, *E. coli* CBW 12241(*ΔiclR*, P_T5_-*appY*), produced lycopene at 33.43 mg per gram of dry cell weight.

**Conclusions:**

A lycopene hyper-producer *E. coli* strain that does not carry a plasmid or antibiotic marker was constructed using triclosan-induced chromosomal evolution. The methods detailed in this study can be used to engineer *E. coli* to produce other metabolites.

## Background

Carotenoids are a diverse class of C_40_ isoprenoids that have multiple physiological and nutritional functions in many organisms. Carotenoids have received considerable attention from the food industry, medicine and cosmetics because of their interesting pigment properties, and more importantly, their potential beneficial effects on human health. Lycopene is an effective antioxidant [1] and has beneficial biological and pharmaceutical activities, including anti-cancer [2], anti-inflammatory [3], and antioxidative activities [4]. Lycopene is widely used as a supplement in functional foods, animal feed, nutraceuticals, pharmaceuticals and as an additive in cosmetics.

Potential commercial applications mean that efficient biotechnological production of lycopene has become increasingly necessary, and a number of reports have described its production using metabolic engineering [5-21]. However, all of these above studies focused on the deletion of undesirable genes and plasmid overexpression of key genes. Such plasmid-based expression systems have several drawbacks, including structural instability, segregational instability or allele segregation [22-24]. These plasmid instabilities cause genetic instability, which reduces the production of the compound of interest. Tyo et al. reported that plasmid-carrying strains lost poly-3-hydroxybutyrate (PHB) productivity after 40 generations with antibiotics [25]. Moreover, antibiotic resistance genes are the most commonly used markers for selecting and maintaining plasmids in hosts during cultivation. However, antibiotics are both costly and banned from food and pharmaceutical production processes. There is also a potential risk of the spread of antibiotic-resistant marker to other microbes in nature, leading to the rapid emergence of multidrug-resistant organisms (e.g., superbacteria) [26,27].

These drawbacks of using plasmids can be overcome by integration of genes into the chromosome. Recently, Tyo et al. developed a plasmid-free method for the over-production of metabolites that can achieve high copy numbers of the desired genes, termed chemically induced chromosomal evolution (CIChE) [25]. Genes of interest were inserted into chromosome of *Escherichia coli* by the λInCh genomic integration method, and then evolved to the desired gene copy number by chemical induction. However, the λInCh genomic integration protocol is complicated and time-consuming, because it contains three steps that involve two recombination steps. Chiang et al. modified the conditional-replication, integration, and modular plasmid system produced by Haldimann and Wanner [28], and developed a replicon-free and markerless method (RMM) for the chromosomal insertion of genes [26]. Genes of interest can be directly integrated into the bacterial attachment site of the *E. coli* chromosome as single copies, through transformation. However, the CIChE strains reported by Tyo et al. still have an antibiotic resistance marker (chloramphenicol resistance) [25]. To avoid the use of antibiotic resistance genes and antibiotics, Goh and Good developed a novel system using the widely used the biocide triclosan as the selective agent and the essential growth gene *fabI* of *E. coli* as the selective marker [27]*.* Thus, to overcome the drawbacks of CIChE as originally developed by Tyo et al., we developed a series of integration expression vectors, pXKF3T5b, for triclosan induction chromosomal evolution in our previous paper [29]. Using these vectors, genes of interest can be inserted into *E. coli* site-specifically by transformation using RMM. The gene copy number can then be evolved to the desired value by triclosan induction. In this study, we constructed a lycopene hyper-producer *E. coli* that does not carry plasmid or antibiotic marker, using the CIChE integration expression vector. To the best of our knowledge, this is the first report of metabolic engineering of an *E. coli* that does not carry a plasmid or antibiotic marker using a multiple gene expression system for lycopene production.

## Results and discussion

### *CIChE of* E. coli *for lycopene production*

To overcome the drawbacks of CIChE as originally devised by Tyo et al., we developed a series of integration expression vectors, pXKF3T5b, for triclosan induction chromosomal evolution in our previous paper [29]. Using these vectors, genes of interest can be inserted into *E. coli* site-specifically by transformation using RMM. The gene copy number can then be evolved to the desired value by triclosan induction. Thus, strains constructed using our CIChE integration expression vectors have no antibiotic resistance and are environmentally safe.

**Figure 1:**
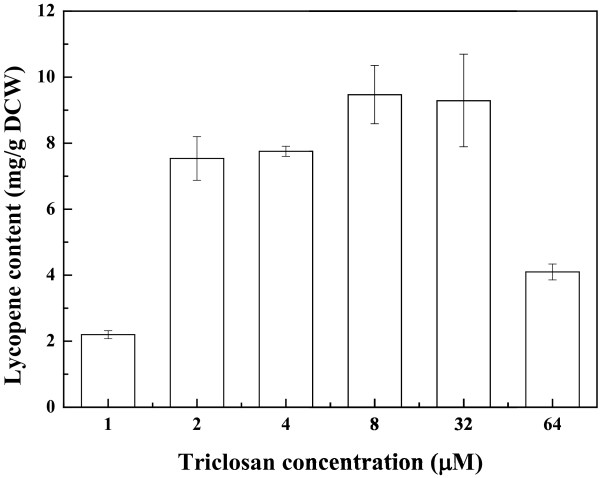
Lycopene production of CIChE strains at different triclosan concentrations cultured in 2YT medium supplemented with 5 g/L KAc.

**Figure 2:**
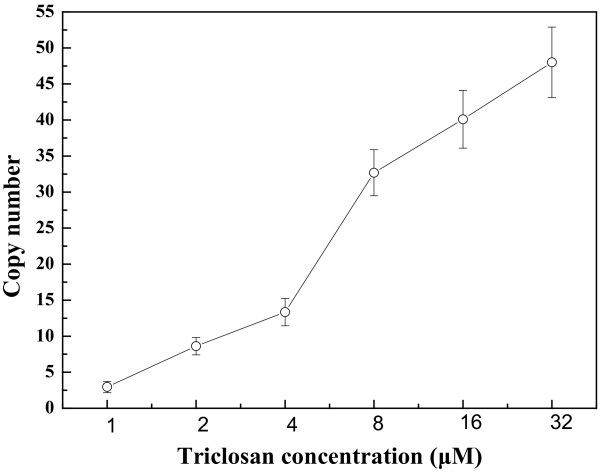
**Copy number of the *crtI *gene in CIChE strains at different triclosan concentrations.**

The CIChE integration expression vector containing the lycopene biosynthetic gene cluster (pP21KF3T5b-EIBipi) was transferred into *E. coli* BW25113 (*ΔgdhAΔaceE*, P_T5_*dxs*) and then increasing triclosan concentration was used to induce CIChE. Figure 1 shows the results of lycopene production in CIChE strains resistant to different triclosan concentrations. Lycopene production in CIChE strains increases with increasing triclosan concentration during chromosomal evolution. The maximum lycopene production of 9.55 ± 0.23 mg/g dry cell weight (DCW) was obtained by the CIChE strains resistant to 8 μM triclosan cultured in 2YT medium. Figure 2 shows that the *crtI* gene copy number of the CIChE strains increases with triclosan concentration during chromosomal evolution. At a triclosan concentration of 8 μM, the copy number reached about 30 in the CIChE strains, which is the equivalent copy number of a medium to high copy plasmid. When the triclosan concentration was above 8 μM, the gene copy number still increased; however, the lycopene production of the CIChE strains did not increase. The results indicated that there is an optimal copy number of the *crt* genes for efficient production of lycopene. Thus, the *recA* gene of the CIChE strain that was resistant to 8 μM triclosan was deleted to obtain *E. coli* CBW12241, in which homologous recombination, which could reduce the copy number, is inhibited. The *crt* gene number of the *recA-*deleted strain did not change (data not shown). The genetic stability was also assayed (Table 1). The level of lycopene production in *E. coli* CBW12241 remained constant after 30 rounds of subculturing without triclosan. However, the plasmid-bearing strain *E. coli* BW25113 (*ΔgdhAΔaceE,* P_T5_*dxs*, pBAD24-WZM1) lost lycopene productivity sharply, whether cultured with antibiotics or not. With antibiotics, the level of lycopene production in the plasmid-bearing strain, *E. coli* BW25113 (*ΔgdhAΔaceE,* P_T5_*dxs*, pBAD24-WZM1), was only about 30% of that of the parent strain after 30 rounds of subculturing. Without antibiotics, the lycopene productivity of the plasmid-bearing strain *E. coli* BW25113 (*ΔgdhAΔaceE,* P_T5_*dxs*, pBAD24-WZM1) dropped to zero after 30 rounds of subculturing. These results are consistent with previous reports wherein the gene copy number and PHB production of a CIChE strain remained constant after 40 rounds of subculturing [25]. They also found that PHB productivity of the plasmid-carrying strain cultured with antibiotics was completely lost after 40 generations. The loss of productivity in the plasmid system with antibiotics may be due to allele segregation. It is likely to occur regularly, despite antibiotic selection, particularly in subculturing experiments or in chemostats [25].

**Table 1:** **Genetic stability of the CIChE and the plasmid-carrying strain**^**a**^

**Number of subculture**	**OD**_**600**_	**Specific lycopene content (mg/gDCW)**
*E. coli* CBW12241		
1	6.85 ± 1.83	28.57 ± 0.50
5	5.59 ± 0.31	29.76 ± 0.76
10	4.37 ± 0.49	31.05 ± 1.11
15	4.47 ± 0.37	30.37 ± 1.21
20	4.61 ± 0.45	30.95 ± 1.21
25	4.39 ± 0.39	28.95 ± 0.85
30	4.92 ± 0.10	30.53 ± 1.53
*E. coli* BW25113 (*ΔgdhA*Δ*aceE*, P_T5_-*dxs,* pBAD24-WZM1)		
1	4.29 ± 0.51	7.51 ± 0.76
30, with Amp	7.15 ± 0.38	2.24 ± 0.46
30, without Amp	8.51 ± 0.45	0

^*a*^ Cells were cultured in SBMSN medium supplemented with 5 g/L KAc without triclosan at 37°C for 48 h. Data represent means of triplicate cultures ± standard deviation.

### Optimization of the lycopene synthetic pathway

Overexpression of the *idi*, *dxs*, *pck*, *pps*, *rpoS*, *appY*, *yjiD*, *ycgW*, *wrbA* and *atpE* genes are reported to improve lycopene production in *E. coli*[8-19,21]. Thus, the effects of over-expression of these genes in *E. coli* CBW12241 on lycopene production were investigated. The results are presented in Table 2. Surprisingly, only the over-expression of *appY* slightly enhanced the specific lycopene content. This may reflect the different genetic backgrounds of the strains. In *E. coli* CBW12241, isopentenyl diphosphate isomerase is overexpressed by chromosomal evolution of *ipiHPI*. Thus, overexpression of the *idi* gene in *E. coli* CBW12241 was not beneficial to lycopene production. Zhao et al. [30] reported that the *dxs* gene from *Bacillus subtilis* functioned more efficiently to enhance isoprene production in *E. coli* than the native *dxs* gene. However, overexpression of the *dxs* gene from *Bacillus subtilis* significantly inhibited lycopene production in *E. coli* CBW12241, possibly because the T5 promoter has replaced the native promoter of the *dxs* gene in *E. coli* CBW12241.

**Table 2:** Effect of plasmid-based overexpression of genes on lycopene production in the CIChE strain *E. coli *CBW12241^*^

**Plasmid**	**OD**_**600**_	**Lycopene concentration (mg/L)**	**Specific lycopene content (mg/gDCW)**
pBAD24	9.54 ± 0.73	57.79 ± 1.60	19.01 ± 1.64
pBappY	8.95 ± 0.08	57.75 ± 0.49	20.16 ± 0.18
pBidi	10.20 ± 0.22	62.49 ± 1.43	19.15 ± 0.66
pBpck	10.28 ± 0.46	58.87 ± 2.34	17.90 ± 0.15
pBpps	10.51 ± 0.57	60.11 ± 0.97	17.91 ± 1.29
pBrpoS	10.91 ± 0.34	60.17 ± 1.54	17.25 ± 0.99
pBycgW	10.53 ± 0.42	60.43 ± 0.92	17.96 ± 0.54
pByjiD	10.54 ± 0.10	58.29 ± 0.34	17.28 ± 0.07
pBdxs	10.25 ± 0.45	62.02 ± 1.66	18.91 ± 0.47
pQE30	7.72 ± 0.13	52.92 ± 1.53	21.42 ± 0.67
pQwrbA	13.76 ± 0.47	7.51 ± 0.58	1.71 ± 0.13
pQydeO	13.19 ± 0.17	9.81 ± 0.62	2.33 ± 0.15
pQatpE	9.73 ± 0.17	45.21 ± 0.67	14.53 ± 0.06
pQdxs	11.49 ± 0.31	25.62 ± 1.84	6.97 ± 0.64

^*a*^ Cells were cultured in SBMSN medium supplemented with 5 g/L KAc without triclosan at 37°C for 48 h. Data represent means of triplicate cultures ± standard deviation.

To reduce the metabolic burden caused by the plasmid, the native promoter of the *appY* gene was replaced with the T5 promoter to obtain *E. coli* CBW12241 (P_T5_*appY*). As shown in Table 3, the replacement improved cell growth and lycopene concentration. Kang et al. used a shotgun approach and found that overexpression of the *appY* gene improved lycopene production [12]. The *appY* gene encodes a transcriptional activator of two regulator energy metabolism operons, *hya* and *cbdAB*-appA, which are induced by anaerobiosis. They reasoned that the *appY* gene might help rescue the strain from energy insufficiency caused by lower ubiquinone levels.

**Table 3:** **Lycopene production and biomass yield of recombinant strains**^**a**^

**Strain**	**OD**_**600**_	**Lycopene concentration (mg/L)**	**Specific lycopene content (mg/gDCW)**
*E. coli* CBW12241	5.87 ± 0.67	54.15 ± 3.44	28.97 ± 0.67
*E. coli* CBW12241 (P_T5_-appY)	8.56 ± 1.50	79.17 ± 7.20	29.59 ± 0.81
*E. coli* CBW12241 (△iclR)	5.38 ± 0.16	51.32 ± 2.86.	29.82 ± 0.78
*E. coli* CBW12241 (△iclR, P_T5_-appY)	7.49 ± 1.58	77.85 ± 1.42	33.43 ± 0.81
*E. coli* CBW12251	6.90 ± 1.01	70.07 ± 1.44	31.73 ± 0.48

^*a*^ Cells were cultured in SBMSN medium supplemented with 5 g/L KAc without triclosan at 37°C for 48 h. Data represent means of triplicate cultures ± standard deviation.

A comparative transcriptome and proteome analysis demonstrated that the deletion of the transcriptional repressor *iclR* of the glyoxylate pathway increased lycopene production [31]. Thus, we deleted the *iclR* gene and examined lycopene production. The deletion of the *iclR* gene in *E. coli* CBW12241 caused a slight inhibition of growth and did not affect the specific lycopene content. The deletion of the *iclR* gene in the promoter replacement strain, *E. coli* CBW12241 (P_T5_*appY*), slightly improved specific lycopene content, from 28.97 ± 0.67 mg/g DCW to 33.43 ± 0.81 mg/gDCW, and improved the lycopene concentration from 54.15 ± 3.44 mg/L to 77.85 ± 1.42 mg/L (Table 3). In the *iclR* gene knockout strain, the glyoxylate pathway is constitutively active [32]. Furthermore, there are other examples of metabolic engineering involving deleting *iclR* to increase productions of compounds. Sanchez et al. successfully knocked out *iclR* to boost the glyoxylate pathway flux for succinate overproduction [33]. Lee et al. observed *aceBA* upregulation in the threonine-overproducing *E. coli*, and when *iclR* deleted, a 30% higher threonine production was recorded [34].

Many reports have been published about lycopene production using recombinant *E. coli*[5-21]. However, these strains contain plasmids, which can cause genetic instability and can have a negative environmental impact. Chiang et al. [26] constructed a lycopene hyper-producer *E. coli* strain that did not carry the replicon or a selective marker using RMM. However, RMM is a single gene copy expression system. Tyo et al. [25] constructed a lycopene producer *E. coli* using CIChE, a plasmid-free, high gene copy expression system. However, this strain still contained a chloramphenicol resistance marker. In the present study, we engineered a lycopene producer *E. coli* that does not carry a plasmid or an antibiotic marker. The CIChE strain only contains the *fab I* gene, which is essential for the growth of *E. coli*. The strain does not present the safety problems associated with antibiotic resistance genes in plasmids. Moreover, the strain does not require the presence of the resistance compound (triclosan) during the fermentation process. To the best of our knowledge, this is the first report of engineering an *E. coli* that does not carry a plasmid or antibiotic marker using a high gene copy expression system.

The maximum specific lycopene content (33.43 mg/g DCW) of *E. coli* CBW12241 (*ΔiclR*, P_T5_*appY*) was higher than that of the CIChE strain reported by Tyo et al. (about 11 mg/gDCW) [25]. The value was also higher than other plasmid-carrying strains, which achieved 22 mg/g DCW [21], 18 mg/g DCW [6] or 32 mg/g DCW produced by the engineered *E. coli* containing heterologous lycopene and mevalonate pathways [35]. However, the yield is slightly lower than that reported by Chiang et al. (38.5 mg/g DCW) [26]. In their study, the RMM strain *E. coli* BL21-CrtD1K has an additional chromosomal copy of *dxs* fused to the T7 promoter. However, plasmid-based overexpression of the *dxs* gene in the CIChE strain did not further enhance lycopene production. Moreover, *E. coli* CBW12251, containing an additional chromosomal copy of the *dxs* gene, produced lycopene at 31.73 ± 0.48 mg/gDCW, which was lower than *E. coli* CBW12241 (*ΔiclR*, P_T5_*appY*) (Table 3).

Although the yield of our engineered *E. coli* harboring only a heterologous lycopene pathway achieved the advanced levels quoted in the literature, many papers have reported that the introduction of a heterologous mevalonate pathway improved production of isoprenoids such as lycopene [16,17,20,35], CoQ_10_[36], α-farnesene [37], terpenoid [38], taxol [39] and amorphadiene [40]. Thus, integration of a heterologous mevalonate pathway into the chromosome of the engineered *E. coli* CBW12241 (*ΔiclR*, P_T5_*appY*) may further improve lycopene production.

## Conclusions

We constructed a lycopene producer *E. coli* strain that does not carry a plasmid or antibiotic marker using CIChE, replacement of a promoter as well as knockout of a gene. No resistance compound was required during the fermentation process using this strain. This is the first report of engineering an *E. coli* that lacks a plasmid and an antibiotic marker using a high gene copy expression system. The engineered strain remained stable, as determined by its lycopene production, after 30 sequential transfers.

## Methods

### Strains, primers and plasmids

The strains and plasmids used in this study are listed in Table 4. *E. coli* DH5α was used for plasmid construction. *E*. *coli* BW2511 (*ΔaceFΔgdhA*, P_T5_*dxs*) [19] was used as the parent strain for chromosomal integration. The primers used in this study are listed in Table 5.

**Table 4:** List of bacterial strains and plasmids used in this study

**Strain or plasmid**	**Description**^**a**^	**Source or reference**
Strains		
*E. coli* DH5α	*supE44* Δ(*lacZYA-argF*) *U169* (Φ*80lacZ* Δ*M15*) *hsdR17 recA endA1 gyrA96 thi-1 relA1*	Invitrogen
*E. coli* BW25113	*lacI*^q^*rrnB*_T14_*ΔlacZ*_WJ16_*hsdR514 ΔaraBAD*_AH33_*ΔrhaBAD*_LD78_	42
*E. coli* BW25113 (*ΔgdhAΔaceE*, P_T5_-*dxs*)	*E. coli* BW25113, *ΔgdhA*, *ΔaceE*, replacement of native promoter of the *dxs* gene with T5 promoter	19
*E. coli* CBW12241	CIChE strain of *E. coli* BW25113 (*ΔgdhAΔaceE*, P_T5_-*dxs*) resistance to 8 μM triclosan, *ΔrecA*	This study
*E. coli* CBW12241 (P_T5_-*appY*)	*E. coli* CBW12241, replacement of native promoter of the *appY* gene with T5 promoter	This study
*E. coli* CBW12241 (*ΔiclR*)	*E. coli* CBW12241, *ΔiclR*	This study
*E. coli* CBW12241 (*ΔiclR*, P_T5_-*appY*)	*E. coli* CBW12241(P_T5_-*appY*), *ΔiclR*	This study
*E. coli* CBW12251	*E. coli* CBW12241 (*ΔiclR*, P_T5_-*appY*) with an additional chromosomal copy of *dxs* under the control of T5 promoter	
Plasmid		
pP21KF3T5b	CIChE integration expression vector, Kan^r^	29
pP21KF3T5b-crtEBIipi	pP21KF3T5b derivative containing *crtE, crtB* and *crtI* gene from *Pantoea agglomerans* and *ipiHPI* from *Haematococcus pluvialis*, Kan^r^	This study
pAH121	Helper plasmid expressing phage P21 Int, Amp^r^	28
pBAD24-WZM1	pBAD24 derivative containing *crtE, crtB* and *crtI* gene from *Pantoea agglomerans* and *ipiHPI* from *Haematococcus pluvialis*, Amp^r^	19
pSIM6	pSC101 replicon^ts^ P_L_*-gam-bet-exo cI*857, Amp^r^	43
pKD4	*oriRγ*, *FRT*::*kan*::*FRT* template plasmid, Kan^r^, Amp^r^	42
pCP20	pSC101 replicon^ts^ Flp(λR*p*) *cI*857, Cm^r^, Amp^r^	42
pBAD24	pMB1 *ori*, P_BAD_ L-arabinose inducible, Amp^r^	41
pBappY	pBAD24 derivative containing the *appY* gene from *E. coli*, Amp^r^	This study
pBidi	pBAD24 derivative containing the *idi* gene from *E. coli*, Amp^r^	This study
pBpck	pBAD24 derivative containing the *pck* gene from *E. coli*, Amp^r^	This study
pBpps	pBAD24 derivative containing the *pps* gene from *E. coli*, Amp^r^	This study
pBrpoS	pBAD24 derivative containing the *rpoS* gene from *E. coli*, Amp^r^	This study
pBycgW	pBAD24 derivative containing the *ycgW* gene from *E. coli*, Amp^r^	This study
pByjiD	pBAD24 derivative containing the *yjiD* gene from *E. coli*, Amp^r^	This study
pQE30	ColE1 *ori*, P_T5_ IPTG inducible, Amp^r^	Qiagen
pQEwrbA	pQE30 derivative containing the *wrbA* gene from *E. coli*, Amp^r^	This study
pQydeO	pQE30 derivative containing the *ydeO* gene from *E. coli*, Amp^r^	This study
pQatpE	pQE30 derivative containing the *atpE* gene from *E. coli*, Amp^r^	This study
pQdxs	pQE30 derivative containing the *dxs* gene from *Bacillus subtilis*, Amp^r^	This study

^a^Amp^r^: ampicillin resistance; Cm^r^: chloramphenicol resistance; Kan^r^: kanamycin resistance; *crtE:* geranylgeranyl diphosphate synthase; *crtB:* phytoene synthase*; crtI:* phytoene desaturase*; ipiHPI:* isopentenyl diphosphate isomerase from *Haematococcus pluvialis; appY*: DNA-binding transcriptional activator; *idi:* isopentenyl diphosphate isomerase; *pck*: phosphoenolpyruvate carboxykinase; *pps:* phosphoenolpyruvate synthetase; *rpoS:* RNA polymerase sigma 38; *ycgW:* inhibitor of σ^S^ proteolysis; *yjiD:* inhibitor of σ^S^ proteolysis; *wrbA:* NAD(P)H: quinone oxidoreductase; *ydeO:* DNA-binding transcriptional dual regulator; *atpE:* ATP synthase, F0 complex, c subunit; *dxs:* 1-deoxy-D-xylulose-5-phosphate synthase.

**Table 5:** Primers used in this study

**Primers**	**Sequence and purpose**^**a**^
BLP1	5^′^- ATCGCCTGTATGAACCTG -3^′^, Diagnostic PCR in *attP*_P21_ integration
BLP4	5^′^- TAGAACTACCACCTGACC -3^′^, Diagnostic PCR in *attP*_P21_ integration
AHP2	5^′^- ACACTTAACGGCTGACATGG -3^′^, Diagnostic PCR in *attP*_P21_ integration
AHP3	5^′^- AACGAGTATCGAGATGGCAC -3^′^, Diagnostic PCR in *attP*_P21_ integration
CGA	5^′^- TCAAGAATCTGGTGACCGAGGAG -3^′^, Diagnostic PCR in *attP*_P21_ integration
CGB	5^′^- ACGCCGCTTCAATGACGCTG -3^′^, Diagnostic PCR in *attP*_P21_ integration
VCA1	GTCGTCAGGCTACTGCGTATG. Diagnostic PCR in *recA* deletion
VCA2	CACGATCCAACAGGCGAG. Diagnostic PCR in *recA* deletion
NDF	5^′^- TGGTAATAATGGCTTCGTCTG -3^′^, qPCR for the *minD* gene
NDR	5^′^- GCGATAAAGATGCCCTCAC -3^′^, qPCR for the *minD* gene
QCF	5^′^- CCAGGAGGGATATTTGC -3^′^, qPCR for the *crtI* gene
QCR	5^′^- CAGGGAGTGGAACGAGAAG -3^′^, qPCR for the *crtI* gene
wrbAR	5^′^*-* CGGTCGACGCGTATCCTCCTGTTGAAGATTAG CCGTT -3^′^
yedeOF	5^′^*-* GCGTCGACAGGAGAGATAAAATGTCGCTCGT TTGTTCT -3^′^
ydeOR	5^′^- GGTCTCTGCAGTCAAATAGCTAAAGCATTCATCGT -3^′^
atpEF	5^′^- GAGAGCTCAGGAGGACTGTCATGGAAAACCTGAAT -3^′^
atpER	5^′^- CGGGGTACCTAAATAAAAGCAACGCTTACTACGC -3^′^
dxsF	5^′^-CTGGGATCCAGGAGATCCGCTATGGATCTTTTATCAATACAGGAC-3^′^
dxsR	5^′^- CCGGGTACCGCTGTCATGATCCAATTCCTTTATGT -3^′^
idiF	5^′^-GAGGTACCAGGAGTTGTTCGATGTCCAACAATG-3^′^
idiR	5^′^-GGCTCTAGATTATTTAAGCTGGGTAAATGCAG-3^′^
appYF	5^′^**-**GCCTGCAGAGGAGGTGCAAGATGGATTATGT-3^′^
appYR	5^′^-CCGCATGCTTATCAGTCAATTGTTTTG-3^′^
pckF	5^′^-GGAATTCTCAATGCGCGTTAACAATGGTT-3^′^
pckR	5^′^*-*CTCCCATGGTTACAGTTTCGGACCAGCC-3^′^
ppsF	5^′^-GAGGTACCAGGAGTTGTTCGATGTCCAACAATG-3^′^
ppsR	5^′^-CGTCCCGGGTTATTTCTTCAGTTCAGCCAGG-3^′^
rpoSF	5^′^-GCCTCTAGAAGGAGCCACCTTATGAGTCAGAATAC-3^′^
rpoSR	5^′^-GGTCGACTTACTCGCGGAACAGCGCTT-3^′^
ycgWF	5^′^-CGGCATGCAGGAGATAACAAATGAAGTGGATAGT-3^′^
ycgWR	5^′^-GCCAAGCTTTAGCATATCGAGCATATTT-3^′^
yjiDF	5^′^-GGTCGACAGGAGGCGCAAAATGATGCGACAAT-3^′^
YJIDR	5^′^-GGCTGCAGTTAGCTGACATTCTCCAGCG-3^′^
APFP	5^′^- CTCCGTATAGAGTTCCATCGT -3^′^, Diagnostic PCR in the replacement of *appY* promoter
APRP	5^′^- GCCACATTTCTGGGCTACGAC -3^′^, Diagnostic PCR in the replacement of *appY* promoter
IKFP	5^′^- TGTTTATCAAGAGTGTCTGAGCGT -3^′^, Diagnostic PCR in the deletion of the *iclR* gene
IKRP	5^′^- CGTTTTCACCGCAAATACCG -3^′^, Diagnostic PCR in the deletion of the *iclR* gene

^a^ Restriction enzyme sites are underlined.

### Chemically induced chromosomal evolution

**Figure 3:**
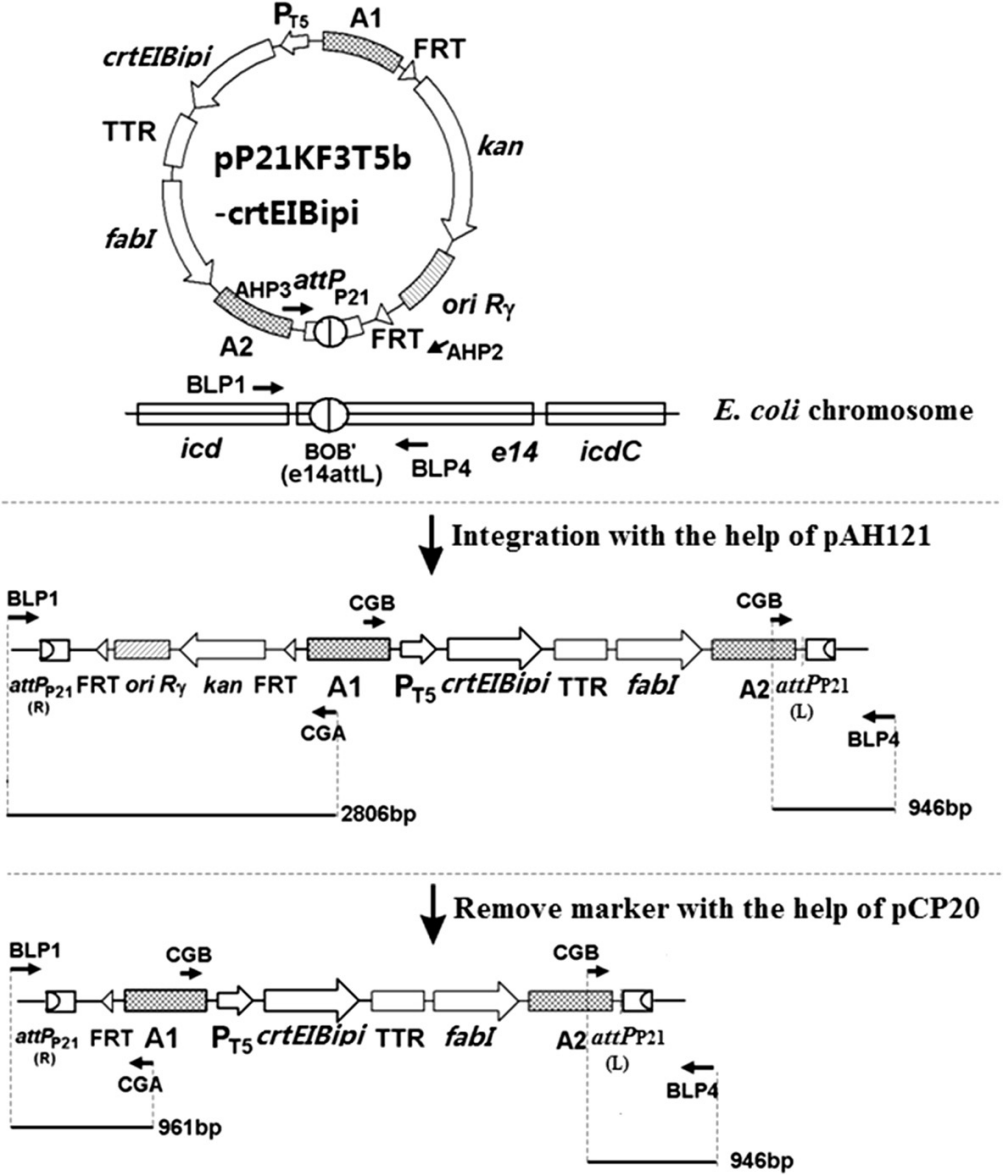
**Change in genomic structure induced by integration of pP21KF3T5b-crtEIBipi at the *attP*_P21_ site.** Primers BLP1, BLP4, CGA, CGB (Table 5) were used to verify the genomic structure by colony PCR. The numbers beside the bars represent the expected sizes of PCR fragments amplified using corresponding primer sets (shown by arrows).

The *crtE*, *crtI*, *crtB* and *ipi* gene cluster was cut from pBAD24-WZM1 [19] using restriction enzymes *ClaI* and *HindIII* and treated with Primestar HS DNA polymerase (TaKaRa, China) and digested with *Eco*RI. The resulting gene cluster was cloned into the *Eco*RI/*Bam*HI sites of pP21KF3T5b [29] to obtain pP21KF3T5b-crtEIBipi. The resulting integration vector was inserted into the bacterial attachment (*attB*) site of *E. coli* using a helper plasmid, pAH121, expressing the phage integrase, by direct transformation, as described by Chiang et al. [26]. The general procedure for using the integration vector is illustrated in Figure 3. In brief, strains containing the helper plasmid pAH121 were first cultured in super optimal broth (SOB) medium with 100 μg/mL of ampicillin (Amp) at 30°C to an optical density at 600 nm of approximately 0.6. The cells were made electrocompetent and transformed with the integration vector pP21KF3T5b-crtEIBipi. Following electroporation, the cells were suspended in SOB plus glucose medium without Ampicillin, incubated at 37°C for 1 h and at 42°C for 30 min, and then spread onto Luria-Bertani (LB) agar plates containing 25 μg/mL of kanamycin (Kan) and incubated overnight at 37°C. Colonies were verified by colony PCR using primer sets BLP1 and CGA, or BLP4 and CGB (Table 5). Positive colonies were cultured in 5 mL LB medium with 25 μg/mL kanamycin at 42°C overnight and then spread onto an LB medium with Kan agar plate at 37°C overnight. Stable integration was scored by antibiotic resistance (conferring Kan resistance) and loss of the helper plasmid (conferring Amp sensitivity), as well as by colony PCR using primer sets BLP1 and CGA, or BLP4 and CGB (Table 5). To eliminate the region containing the selective marker (Kan resistance) and the replication origin, the resulting integrants bearing the inserted DNA were transformed with pCP20 expressing FLP recombinase [23]. Colonies only resistant to triclosan were verified by colony PCR using primer sets BLP1 and CGA, or BLP4 and CGB.

CIChE of the above construct was carried out by subculturing the resulting strains in 5 mL SOB medium with increasing concentrations of triclosan, in 15 mL culture tubes, as described by Tyo et al. [25]. The strains were grown to stationary phase in 1 μM triclosan. Fifty milliliters of the culture was subcultured into a new culture tube. In the new tube, the triclosan concentration was doubled from 2 μM to 64 μM and allowed to grow to stationary phase. The process was repeated until the desired concentration was reached. The *recA* gene of the CIChE strain was then deleted (see below).

### Construction of the plasmid

The *appY*, *idi*, *pck*, *pps*, *rpoS*, *ycgW* and *yjiD* genes were amplified from *E. coli* genomic DNA using the primers shown in Table 5, and ligated into pBAD24 [41], respectively. The *wrbA*, *ydeO* and *atpE* genes were amplified from genomic DNA of *E. coli* using the primers shown in Table 5 and ligated into pQE30, respectively. The *dxs* gene was amplified from genomic DNA of *B. subtilis* ATCC 6633 using primers dxsF and dxsR (Table 5) and ligated into pQE30 (Qiagen).

### Knockout of genes

Gene knockouts and replacement of the native promoter of the *appY* gene with the T5 promoter were carried out by PCR product recombination [42] using the pSIM6 plasmid [43] expressing the lambda red recombination system and pKD4 [42] as the template for PCR. Gene knockouts were verified using colony PCR using the appropriate primers (Table 5).

### Quantitative PCR (qPCR) measurement of gene copy number

Gene copy numbers were measured by qPCR on genomic DNA isolated from the appropriate CIChE strains. qPCR was performed with an iCycler iQ5 Real Time PCR system (Bio-Rad Laboratories, USA) using the SYBR Premix Ex Taq II (TaKaRa, China), following the manufacturer’s protocol. PCR conditions were as follows: 30 s at 95°C; 40 cycles of at 95°C for 5 s, 60°C for 30 s, and 95°C for 60 s; followed by melting curve analysis. The copy numbers of the *crtI* gene were detected and compared to the copy number of *minD*, a nearby native gene in the chromosome. The primers QCF and QCR (Table 5) were used to measure the copy number of *crtI*. The primers NDF and NDR (Table 5) were used to measure the copy number of *minD*.

### Lycopene production

For lycopene production, 5 mL of LB medium supplement with 5 g/L KAc was used for overnight precultivation of *E. coli* in a falcon tube at 37°C. The main cultures were in 2YT medium or SBMSN medium supplemented with 5 g/L KAc. SBMSN medium (pH7.0) contains (g/L): peptone 12, yeast extract 24, KH_2_PO_4_ 1.7, K_2_HPO_4_ 11.42, MgCl_2_·6H_2_O 1, ammonium oxalate 1.42, Tween-80 2. The main cultures were inoculated with a starting OD_600_ of 0.1 and incubated at 37°C for 24 h in a rotary shaking incubator at 150 rpm. Cell growth was measured by optical density at 600 nm and converted into DCW (g/L) using a standard curve.

### Extraction and measurement of lycopene

The lycopene content of recombinant *E. coli* strains was quantified as previously reported [5]. Two hundred fifty microliters of *E. coli* cells were harvested by centrifugation at 12000 rpm for 5 min. The cell pellet was washed with water and then extracted in 1 mL of acetone at 55°C for 15 min with intermittent vortexing. The mixture was then centrifuged at 12000 rpm for 10 min, and the acetone supernatant was transferred to a new tube. The absorbance of the resulting extract was measured at 474 nm and converted to lycopene concentration (μg/mL) using a standard curve obtained using commercial lycopene (Sigma).

### Statistical analysis

All experiments were conducted in triplicate, and data were averaged and presented as the mean ± standard deviation. One-way analysis of variance followed by Tukey’s test was used to determine significant differences using the OriginPro (version 7.5) package. Statistical significance was defined as *p* < 0.05.

## Competing interests

The authors declare that they have no competing interests.

## Authors’ contributions

YY C carried out most of the experiments. HJ S constructed *E. coli* CBW12251 and investigated the effect of the *dxs* gene. YY C constructed the integration expression vectors. SG C carried out fermentation experiments. ZM W constructed expression vectors for some of the genes. M Z guided the project. JZ L developed the concept and designed the method, led the project and drafted the manuscript. All authors read and approved the final manuscript.
